# O077: The impact of a multi-faceted approach on the reduction of peripheral line-associated MRSA bloodstream infections in a high endemic setting

**DOI:** 10.1186/2047-2994-2-S1-O77

**Published:** 2013-06-20

**Authors:** E Tartari, MA Borg

**Affiliations:** 1 Infection Prevention and Control Unit, Mater Dei Hospital, Msida, Malta

## Introduction

The Mediterranean region is characterized by a high prevalence of methicillin-resistant *Staphylococcus aureus* (MRSA) bloodstream infections (BSI). Following root cause analysis (RCA) of MRSA BSI cases, Mater Dei Hospital, a tertiary care institution in Malta, identified that a 30% of healthcare associated (HA) MRSA BSI from April 2010 to December 2011 were associated with Peripheral Venous Catheters (PVC).

## Objectives

To identify the impact of the implementation of a bundle of measures on the rate of catheter-related BSI.

## Methods

The study involved a baseline phase (08/2010-2/2011) followed by an intervention period (3/2011-12/2011) and a sustainability phase (1/2012-12/2012). During the baseline phase, every HA-MRSA BSI was analysed through a RCA to identify those caused by PVC. At the same time, practices related to PVC insertion and maintenance were audited. During the intervention phase: (1) a new hospital policy, insertion record and maintenance checklist for daily assessment and documentation were launched, (2) Visual Infusion Phlebitis (VIP score) tool and PVC duration not exceeding 72 hrs, (3) hands-on training for junior doctors on insertion and educational sessions for all nursing staff on PVC ongoing care were provided, (4) and the introduction of a semi-permeable transparent dressing. Auditing and feedback with corrective action on non-conformities were undertaken in all of the hospital wards during the sustainability period.

## Results

The number of MRSA BSI cases has halved in our institution, from a yearly average of 1.85 BSI per 10,000 patient days in 2009 to 0.89 infections per 10,000 patient days in 2012.From January 2011 to February 2013, the number of cases have been stratified by yearly quarter. In year 2011, 36 MRSA blood infections with PVC as primary origin were reported. In year 2012, 12 MRSA blood infections were reported in Quarter 1 and 6 cases were reported in Quarter 2. Over the past 9 months the number of HA- MRSA BSI has constantly reduced and dropped to zero cases between July 2012 and February 2013.

## Conclusion

CR-BSIs are essentially preventable. We consider multidisciplinary strategies such as RCA and continuous audits of PVC insertion/maintenance with real time feedback and corrective action to have contributed most to the success.

## Disclosure of interest

None declared.

